# Clinical Characteristics and Main Findings of Colonoscopy in Tripoli Central Hospital: A Cross-Sectional Study of 1858 Patients

**DOI:** 10.7759/cureus.34983

**Published:** 2023-02-14

**Authors:** Osama Naseer, Mahjoub Bashir Rishi, Abdulhakim M Gelia, Khaled Saad Taggaz, Ali Mohammed Zawia, Maram Sadeq Elarifi, Iman Daw Alsaghir

**Affiliations:** 1 General Surgery Department, Tripoli Central Hospital, Tripoli, LBY; 2 General Surgery Department, Tripoli Central Hospital, Tirpoli, LBY

**Keywords:** post-colonoscopy, surveillance colonoscopy recommendations, colonoscopy、 perforation、endoscopic therapy、 retrospective observational study, screening colonoscopy, colonoscopy complications

## Abstract

Introduction

Colonoscopy is used to detect colorectal abnormalities, including inflammatory bowel disease, polyps, cancers, and other colorectal lesions. We aimed to analyze the demographic and clinical characteristics, main findings, and indications of patients who underwent colonoscopy in the Surgery department of Tripoli Central Hospital in Libya.

Methods

The study data were retrospectively extracted from the medical in and out-patient records of individuals who underwent colonoscopy procedures between December 2009 and December 2016 in the general surgery department of Tripoli General Hospital.

Results

A total of 1858 patients underwent colonoscopy during the study period with a mean age of 51.7 ± 18.5 years. Hematochezia was the most common patient complaint (530; 28.5%), followed by constipation (354; 19.1%), and weight loss (178; 9.6%), respectively. Seven-hundred sixty-five (765; 41.2%) participants completed the procedure, 420 (22.6%) did not, and 673 (36.2%) participants failed the colonoscopy. The most common reasons for procedure failure were failed preparation (609; 55.7%), followed by patient intolerance (251; 23.0%), and obstructive lesions (229; 21.0%). The most common finding was colonic masses, followed by polyps (29.0% and 20.8%, respectively).

Conclusion

This study describes the characteristics of colonoscopy patients in the largest surgical center in Libya over seven years. Hematochezia and chronic constipation were the most common complaints among the participants with reported complaints. Half of the colonoscopy procedures are incomplete or failed due to the lack of patient preparation. Colonic masses and polyps were the most common among the reported colonoscopic findings. Future research to increase the quality of colonoscopy service and patient preparations in Libya is required.

## Introduction

Colonoscopy is the gold-standard examination of lower gastrointestinal symptoms such as abdominal pain, polyps, diarrhea, difficulty defecation, hematochezia, or mucoid stool. Colonoscopy is performed through a long flexible tube called a colonoscope with a fiber-optic camera at its tip and enters the body through the rectum [1]. It is helpful for the detection and/or removal of precancerous and cancerous colonic or rectal lesions [2]. Colonoscopy is essential for the final diagnosis of inflammatory bowel disease (IBD), colonic adenomas, colonic polyps, and colorectal cancer (CRC) [3]. The progress and development of the modern gastroenterology field depend significantly on the advances in colonoscopy technology [3]. The colonoscopy was completed if the endoscope reached the cecum and ileum or the anastomosis in subjects with surgical resection for the tumor [4].

Libya is a war-torn country despite having large, proven oil reserves: worldwide (3%) and in Africa (39%). Political instability and militia attacks have restricted the country's development due to low external and internal investments since 2011 [5]. A recent study showed that most colorectal cancer patients in Libya present in the late or metastatic stages, which requires further improvements in the screening methods and particularly necessitates more efficient colonoscopy protocols [6]. Another epidemiological study showed that Libya has the highest incidence of CRC in North African countries. In Libya, CRC was the most common cancer among men and the second most common cancer among women after breast cancer [7].

Tripoli, Libya's biggest city and official capital, has over one million citizens out of seven million Libyans [8]. Despite the high safety, acceptability, and efficacy of colonoscopy globally, the applicability, availability, and screening protocols might be limited in some poor-resource settings.

To better understand the demographic and clinical characteristics of colonoscopy patients and colonoscopy procedure characteristics, we conducted this retrospective chart review of all patients who underwent colonoscopy in Tripoli Central Hospital, Libya, within seven years, from 2009 to 2016.

## Materials and methods

We followed the Strengthening the Reporting of Observational Studies in Epidemiology (STROBE) statement guidelines when reporting this manuscript. The ethics committee of Tripoli Central Hospital approved this study. The confidentiality and anonymity of the participants were maintained when exporting these data from the database.

Study setting and design

The study setting was the surgery department of Tripoli General Hospital, the only hospital conducting colonoscopy procedures in the whole of Tripoli. This cross-sectional study aimed to assess the demographic and clinical characteristics, main findings, and indications of patients who underwent the service of the colonoscopy procedure.

Study population

The study data were retrospectively extracted from the medical in and out-patient records of individuals who underwent colonoscopy procedures between December 2009 and October 2016 in the surgery department of Tripoli General Hospital.

Selection criteria

We did not apply strict selection criteria. All patients who underwent colonoscopy within the study period were included in this analysis if they met the following criteria:

(1) Adult patients, age >18 years

(2) Both sexes (males and female)

The procedure of case identification

We retrospectively identified patients from a prospectively collected database in the surgery department of Tripoli Central Hospital. At the time of initiating this study, a total of 1858 patients were eligible for inclusion in the final analysis.

Data collection

We retrieved this study data from the Surgery department of Tripoli Central Hospital, which hosts a prospective database of patient data. On a weekly basis, the surgical team, including surgical specialists, trainees, and registrars, submit the electronic records to Google Drive forms based on the paper copies of the patient tickets. The database was maintained by a computer engineer responsible for database maintenance, coding, and exporting, with no further involvement in the study.

Study variables

The main variables of this study were as follows: sex, age, training level of the medical personnel conducting the colonoscopy, patient complaints, endoscope entry point, endoscopy type, presence, number, and type of pathologies in every patient.

Statistical analysis

Data were summarized and described as frequencies and percentages for categorical variables or mean and standard deviation for continuous variables. The Shapiro-Wilk test was conducted to ensure the normality distribution of the continuous variables. Data were analyzed using Jamovi version 2.0 for macOS (https://www.jamovi.org/).

## Results

Demographics of the study participants

A total of 1858 patients were included in this study. Among the study participants, 928 (49.9%) were males while 930 were females (50.1%). The mean age of the patients was 51.7 (±18.5) years (Table 1).

**Table 1:** The characteristics of the patients and the presenting complaint indicating colonoscopy CA=Cancer; FAP=Familial adenomatous polyposis; SD=Standard deviation

Variable		Descriptives (n=1858)
Sex	Female	930 (50.1%)
	Male	928 (49.9%)
Age	Mean (SD)	51.7 (18.5)
	Range	11.0 - 98.0
Condition indicating colonoscopy	Not reported	51 (2.7%)
	Bleeding per rectum	530 (28.5%)
	Chronic constipation	354 (19.1%)
	Follow post-surgical resection and anastomosis	297 (16.0%)
	weight loss	178 (9.6%)
	Perianal fistula (for further evaluation)	120 (6.5%)
	Follow up CA	120 (6.5%)
	Anal Pain	90 (4.8%)
	Diarrhea	32 (1.7%)
	FAP	27 (1.5%)
	Screening (strong family history)	27 (1.5%)
	Abdominal Pain	16 (0.9%)
	Evaluation of Hartmann's operation	10 (0.5%)
	Ulcerative colitis	6 (0.3%)

Hematochezia was the most common patient complaint among the reported complaints, followed by constipation and weight loss, representing 530 (28.5%), 354 (19.1%), and 178 (9.6%), respectively. Additionally, ulcerative colitis was the least common complaint, reported as the reason for endoscopy in six (0.3%) of the patients (Table 1).

Characteristics of colonoscopy

Most patients underwent colonoscopy (77%). Sigmoidoscopy and proctoscopy accounted for 14.7% and 8.3% of cases. According to the training level of surgeons, cases were operated on by consultant surgeons (56.1%), specialist surgeons (24.5%), or senior house officers (SHOs) (19.4%).

Colonic masses were the most common among the reported colonoscopy findings followed by polyps, representing 213 (29.0%) and 153 (20.8%), respectively (Table 2).

**Table 2:** The endoscopic findings

Variable		n (%)
Endoscope Entry Point	Anus	1826 (98.3%)
	Colostomy	15 (0.8%)
	Both (anus & stoma)	17 (0.9%)
Pathology Found	Not reported	673
	Yes	735 (62.0%)
	No	450 (38.0%)
Number of Pathologies Found	N-Miss	1138
	Single	458 (63.6%)
	3 or more	151 (21.0%)
	Two	80 (11.1%)
	Chronic	31 (4.3%)
Type of Endoscopic Finding	Not reported	1123
	Mass	213 (29.0%)
	Polyp	153 (20.8%)
	Diverticulum	110 (15.0%)
	Piles	65 (8.8%)
	Inflammation	65 (8.8%)
	Fissure	59 (8.0%)
	Ulcer	41 (5.6%)
	Fistula	14 (1.9%)
	Thick mucosa	9 (1.2%)
	Stricture	6 (0.8%)
Site of the Endoscopic Finding	Not reported	1123
	Rectum	217 (29.5%)
	Anal canal	202 (27.5%)
	Sigmoid	174 (23.7%)
	Descending	77 (10.5%)
	Transverse	37 (5.0%)
	Ascending	17 (2.3%)
	Cecum	11 (1.5%)

Procedure outcome, reasons for failure, and next steps

The colonoscopy procedure failed in 673 (36.2%) of the study participants. Seven-hundred sixty-five (765 (41.2%) of the study participants completed the procedure, 420 (22.6%) did not complete the procedure, and 673 (36.2%) failed the procedure. The procedure failure or incompletion was due to multiple factors such as failed preparation, followed by patient intolerance, obstructive lesions, redundant colon, rigid sigmoid, and easily bleeding mucosa, representing 609 (55.7%) of participants, followed by 251 (23.0%), 229 (21.0%), 2 (0.2%), 1 (0.1%), and 1 (0.1%), respectively (Table 3).

**Table 3:** A summary of the outcomes of the procedure and the next steps CT=Computed tomography; MRI=Magnetic resonance imaging; GIT=Gastrointestinal tract; IBD=Inflammatory bowel disease; NPO=Nothing by mouth

Variable		n (%)
The outcome of the procedure	Complete	765 (41.2%)
	Failed	673 (36.2%)
	Incomplete	420 (22.6%)
Reason for failure or incompletion	Not reported	765
	Failed preparation	609 (55.7%)
	The patient does not tolerate	251 (23.0%)
	Obstructed by lesion	229 (21.0%)
	Redundant colon	2 (0.2%)
	Rigid sigmoid	1 (0.1%)
	Easily bleeds mucosa	1 (0.1%)
Step after colonoscopy	Not reported	673
	Repeat Scope	296 (25.0%)
	Admission for medical or surgical treatment	216 (18.2%)
	Fiber diet	210 (17.7%)
	Follow up	120 (10.1%)
	Hemorrhoid ointment	82 (6.9%)
	Enema study	52 (4.4%)
	Laxative	30 (2.5%)
	Advice was written through prescription	20 (1.7%)
	Lidocaine ointment	20 (1.7%)
	Reassurance	20 (1.7%)
	CTs abdomen & pelvis	20 (1.7%)
	Antispasmodic drug	17 (1.4%)
	MRI	15 (1.3%)
	Anti-biotic	12 (1.0%)
	Veno-constrictor tab (Daflon)	10 (0.8%)
	For upper GIT endoscope	10 (0.8%)
	Reverse colostomy	8 (0.7%)
	IBD treatment drugs	6 (0.5%)
	Steroid enema	6 (0.5%)
	Anti-spasm ointment	5 (0.4%)
	Anti-gas tablet	4 (0.3%)
	NPO for 24 hours	2 (0.2%)
	Rectal tube decompression	2 (0.2%)
	Barium enema	2 (0.2%)

Repeat endoscopy was advised for 25% of the patients, 18.2% were admitted to receive surgical or medical treatment, 17.7% were advised fiber diets, and the remaining patients received variable recommendations (Table 3).

## Discussion

Colonoscopy is used for the diagnosis and removal of precancerous and cancerous colonic or rectal lesions, including the diagnosis of inflammatory bowel disease, colonic adenomas, colonic polyps, and colorectal cancer, recently. Moreover, the clinical characteristics, main findings, and indications of colonoscopy have been assessed in different regions of the world, especially in developed countries [9-11].

To the best of our knowledge, this is the first cross-sectional study to assess colonoscopy's clinical characteristics and main findings in Tripoli, Libya. Our results expand the literature by providing new information about the demographic, clinical characteristics, and main findings of colonoscopy procedures in the Surgery department of Tripoli Central Hospital. The mean age of the study participants was 51.7 ± 18.5, and females were slightly higher than males. This study showed that bleeding per rectum and chronic constipation were the most common reasons for colonoscopy. The most common findings were masses and polyps. A significant proportion of patients have an incomplete or failed colonoscopy, mostly because of the failed preparation or the patient's intolerance to the procedure.

As mentioned earlier, the colonoscopy was completed if the endoscope reached the cecum and ileum or the anastomosis in subjects with surgical resection for the tumor. Our results showed a completed colonoscopy in 765 participants (41.2%). Bowles et al. reported colonoscopy completion rates ranging from 56.9 to 76.9% according to the exact definition [10]. Similarly, Qu et al. reported the procedure's completion in 315/448 participants (70.31%) using the precise completion definition [4]. These differences could be explained by the difference in the type of patients who undergo colonoscopy, different settings, and different medical facilities and personnel.

In this study, colonic polyps were found in 20.8% of the participants. In African countries, such as Kenya, Nigeria, and Zimbabwe, colonic polyps have been reported at lower rates, from 5% to 10.3% in several studies [12-14]. A recent retrospective observational study conducted in Tanzania found that colonic polyps occur in 25% of colonoscopy patients [4]. Since some types of colonic polyps are genetically inherited, this difference may be explained by the variations in patient race and origins (e.g., Arabs, Africans, … etc.).

The indications and system of colonoscopy are recently debated topics in Libyan healthcare systems. On the one hand, studies report a high incidence of CRC in Libya compared to other North African countries and a more late presentation with advanced or metastatic CRC [6,7]. On the other hand, there are no national guidelines for colonoscopy procedures in Libya. Each center operates based on physician judgment, expert opinion, and local regulations. A recent study showed that a modified open-access referral system increases the appropriateness of colonoscopy procedures even if no definitive guidelines were followed [15].

Strengths and limitations

To the best of our knowledge, this is the first study to assess the clinical characteristics and main findings of colonoscopy in Tripoli using data from the largest surgical center in Libya. The results of this study reflect the real-life characteristics of colonoscopy patients in Tripoli, Libya, as well as the indications of colonoscopy according to the local guidelines. Also, the political instability in Libya during the study period may play a role in the shortage of medical facilities, especially endoscopic devices, and trained medical personnel, resulting in a decreased proportion of patients receiving a colonoscopy. 

## Conclusions

This study describes the characteristics of colonoscopy patients in the largest surgical center in Libya over seven years. Hematochezia and chronic constipation were the most common complaints among participants with reported complaints. Half of the colonoscopy procedures are either incomplete or failed due to the lack of patient preparation for the procedure. Colonic masses and polyps were the most common among the reported colonoscopic findings. Future research to increase the quality of colonoscopy service and patient preparations in Libya is required.
